# Baseline Findings of an HIV Incidence Cohort Study to Prepare for Future HIV Prevention Clinical Trials in Kisumu, Kenya

**DOI:** 10.3855/jidc.2636

**Published:** 2012-12-15

**Authors:** Wairimu Chege, Sherri L. Pals, Eleanor McLellan-Lemal, Sanjyot Shinde, Monicah Nyambura, Frederick O. Otieno, Deborah A. Gust, Robert T. Chen, Timothy Thomas

**Affiliations:** 1 Centers for Disease Control and Prevention (CDC), Division of HIV/AIDS Prevention, Atlanta, United States; 2 Center for Global Health Research, Kenya Medical Research Institute (KEMRI)/CDC Research and Public Health Collaboration, Kisumu, Kenya

**Keywords:** HIV, Kenya, gender differences, risk behaviors

## Abstract

**Introduction:**

In an analysis of baseline findings of an HIV incidence cohort study, an assessment was made of HIV prevalence among persons presenting for enrollment and any differences in demographic characteristics between persons not enrolled compared to those enrolled. We also described and compared HIV risk behaviors in males and females enrolled in the study.

**Methodology:**

A computer-assisted survey was administered to collect baseline demographic and HIV risk data from 1,277 men and women aged 18–34 years. Testing for HIV and other sexually transmitted infections (STI) was conducted. Out of 1,277 persons prescreened for eligibility, 625 were enrolled.

**Results:**

HIV prevalence of all persons who completed screening was 14.8% (females: 21.1%; males: 8.1%). The odds of being enrolled in the study were higher for persons 18–24 years compared to those 30–34 years of age [adjusted odds ratio (AOR)=2.18, CI=1.13, 4.21] and males compared to females [AOR=2.07, CI=1.43, 2.99]. Among those enrolled in the study, the most prevalent HIV risk behaviors were unprotected sex (49%), alcohol use (45%), and transactional sex (30%) in the last three months. Compared to females, a significantly greater proportion of males reported using any alcohol or recreational drug in the last three months, a history of oral sex, sex with partner other than a spouse or main partner, ever having a blood transfusion, ever being treated for an STI, and having knowledge of their last HIV test result.

**Conclusion:**

The Kisumu Field Station successfully recruited individuals with HIV risk characteristics for the HIV incidence cohort study.

## Introduction

The search for new HIV prevention interventions, such as vaccines, microbicides, pre-exposure prophylaxis, and behavioral interventions, requires conducting randomized controlled clinical trials, the gold standard study design for establishing safety and efficacy for these interventions [1]. Preparation for such trials should target populations in settings of high HIV incidence or prevalence to be able to recruit persons at high risk for HIV and entails the development of the physical site, establishing cohorts of potential trial participants, building staff and laboratory capacity, and creating community awareness and engagement [2]. Experience gained during the process of preparing for cohort studies, especially measuring baseline HIV incidence, is essential to the planning and successful conduct of future trials of new prevention interventions [3–4].

Sub-Saharan Africa continues to be the region most heavily affected by the HIV epidemic. In 2007, the majority (69%) of adults with HIV worldwide lived in sub-Saharan Africa and 72% of AIDS deaths occurred there [4]. The 2007 Kenya AIDS Indicator Survey estimated HIV prevalence among Kenyans aged 15 to 64 years as 7.1%, and identified Nyanza Province in western Kenya as having the highest HIV prevalence (14.9%) among the eight provinces of Kenya [5]. These figures were similar to national (6.7%) and Nyanza Province (15.1%) HIV prevalence estimates among Kenyans age 15 to 49 years [5].

Over the last decade, Kisumu, the administrative capital of Nyanza Province, has been an important location for HIV epidemiologic studies [6] and randomized controlled clinical trials of new interventions including male circumcision [7] and herpes suppression for HIV prevention among discordant couples [8]. As new effective interventions such as male circumcision are implemented and as potential changes in risk behavior may take place over time, it will be necessary to characterize the demographics and risk behavior of populations likely to be targeted for future trials of other HIV prevention interventions, and to document successful recruitment and enrollment of these individuals into HIV studies. Thus the purpose of this study is to describe characteristics of persons recruited and enrolled in an HIV vaccine preparatory cohort.

In January 2007, Kenya Medical Research Institute (KEMRI)/CDC initiated an HIV incidence cohort study to prepare for future community-based HIV vaccine (*e.g*., the planned Partnership for AIDS Vaccine Evaluation or PAVE 100 trial) or other prevention trials among young adults in Kisumu. The present analysis reports on the baseline findings of the cohort study which had the following primary objectives: 1) assess the prevalence of HIV among persons presenting for enrollment, and 2) determine if differences existed in demographic characteristics between persons not enrolled compared to persons enrolled. A secondary objective was to describe and compare HIV risk behaviors in males and females enrolled in the study. This paper will not report on HIV incidence or correlates of HIV infection. These findings will be presented in a separate analysis.

## Methodology

### Study population and study site

The study was conducted in Kisumu, Nyanza Province, which has a population of approximately 500,000 residents that are predominately of Luo ethnicity [9]. The study catchment area comprised the city of Kisumu and bordering districts; a convenience sample approach was used. Criteria for participation included the following attributes: being HIV uninfected men and women (non-pregnant), 18 to 34 years of age who were sexually active within the three months prior to study enrollment and a resident of Kisumu or the surrounding area. The investigation was conducted at the study clinic of the KEMRI/CDC Clinical Research Center (CRC), which is adjacent to the New Nyanza Provincial General Hospital in Kisumu. All blood, urine, and vaginal specimens were processed and tested at the KEMRI/CDC ISO certified laboratory located at the CRC [10].

### Overall study design

We established an observational, prospective cohort beginning January 2007 that involved pre-screening, screening, enrollment and 3-monthly visits for a total of 12 months of study follow-up. Persons who tested HIV positive post enrollment were followed for an additional period of 24 months following HIV positive diagnosis.

### Ethical review

The study protocol, consent forms, and data collection instruments for this study were reviewed and approved by the KEMRI local and national Scientific Steering Committees and national Ethical Review Committee as well as the United States CDC Institutional Review Board.

### Community engagement and recruitment

Community engagement focused on three key activities: establishing a community advisory board (CAB); creating ongoing dialogue and fostering involvement with community stakeholders; and implementing locally appropriate as well as research feasible community mobilization approaches. The CAB was comprised of 15 individuals from key categories in the local community including chiefs, religious leaders, teachers, persons living with HIV, youth, women’s groups, fishermen, local transportation, and other community-based organizations. The CAB served as a primary link between the community and the study staff. In addition to providing recommendations on how best to facilitate community awareness about the research study, the CAB advised on how to approach and negotiate interactions with influential community members, including community leaders, school heads, opinion leaders, and leaders of religious, women’s and youth groups.

Recruitment occurred in multiple venues such as market centers, truck stops, beaches, churches, women’s groups, community groups, formal and informal work-based groups, colleges and schools, and HIV Voluntary Counseling and Testing centers. In addition, we distributed brochures containing basic study information to potential study participants and invited interested persons to visit the study clinic for further information.

We monitored numbers of potential participants coming to the study clinic daily. Due to low enrollment of women during the first two months of the study, recruitment strategies were altered from the first-come-first-served approach used initially to a sex-based quota system to enhance recruitment of women.

### Baseline screening and enrollment procedures

Pre-screening for basic eligibility, informed consent, screening and enrollment procedures were conducted at the KEMRI/CDC CRC study clinic. All data collection instruments and consent documents were available in English and translated in local languages (Kiswahili and Dholuo). Study staff was fluent in all three languages and conducted procedures in each participant’s language of choice.

We performed a brief pre-screening assessment using interviewer-administered computer-assisted personal interview (CAPI) to determine the first level eligibility criteria: engaged in sexual activity in last 3 months, not planning to move in next 12 months, willing to have an HIV test and receive results, not pregnant or plan to become pregnant in next 12 months, not in another HIV intervention study, never tested HIV positive, 18–34 years of age, resident of Kisumu catchment area, and willing to give locator information. Persons who met the pre-screening criteria received detailed information about the study prior to providing written informed consent to proceed with screening procedures.

Screening procedures consisted of a baseline behavioral questionnaire using audio computer-assisted self-interview (ACASI); medical history; a clinical evaluation documented using CAPI; and a sample collection of blood, urine, and vaginal swabs. Male circumcision status was documented clinically. We tested for hemoglobin and platelet levels, liver and kidney function, sexually transmitted infections (STI: syphilis, HSV-2, chlamydia, gonorrhea), and pregnancy for women. Rapid HIV testing was conducted at the initial screening visit with pre- and post-test counseling.

We scheduled an appointment two weeks thereafter to deliver laboratory results and make available the final determination of study eligibility. The complete listing of study inclusion and exclusion criteria is shown in Table 1. If eligible, individuals were enrolled at that visit, issued a study photo identification card with a barcode, and detailed locator information was obtained; this was later verified by a home visit by study staff.

Individuals who tested HIV positive at baseline screening were provided with CD4 count results and referred to HIV care and treatment clinics. All participants received monetary transport reimbursement (300KSh or about $5 US per study visit). No other monetary incentives were provided for study participation.

### Laboratory procedures

Haemoglobin and platelet counts were performed using the BD Coulter Counter (Beckman Coulter Inc., Roissy, France) to obtain a complete blood count from whole blood, while the liver and kidney functions were analyzed using the Cobas Integra 400 plus (Roche Diagnostics, Mannheim, Germany) from serum. Urine pregnancy testing was performed using First Sign HCG One Step (UNIMED International, Inc., South San Francisco, CA, USA). Syphilis testing was conducted using a BD Macro-Vue RPR (Rapid Plasma Reagin) card test (BD & Company, Baltimore, USA) card test and all reactive tests were confirmed by Serodia TP-PA Syphilis Test (Fujirebio Inc, Tokyo, Japan).

HSV-2 was screened using HSV-2 IgG enzyme-linked immunoassay (ELISA) and infection with Chlamydia trachomatis or Neisseriae gonorrhoeae was evaluated by qualitative polymerase chain reaction, using COBAS AMPLICOR CT/NG (Roche Diagnostics, Mannheim, Germany). Real-time parallel rapid HIV testing was conducted using Uni-Gold HIV-1/2, (Trinity Biotech, Wicklow, Ireland) and Determine HIV-1/2 (Abbott Labs, Tokyo, Japan) with Bioline (Meridian Life Science Company, Cincinnati, Ohio) used as a tie-breaker.

### Analysis

To describe the sample and to compare enrolled males and females we computed frequencies, medians and Chi-square statistics. Because expected cell counts for some variables dropped below 5, we used Fisher’s exact test for all comparisons. A Wilcoxon test was used for continuous variables. In addition, we conducted bivariate and multiple regression analyses to determine demographic variables associated with being enrolled in the study or not. All demographic variables, regardless of their significance in bivariate analysis, were entered into the multiple logistic regression model. For HIV prevalence estimates, we calculated rates overall and by age and gender strata. Confidence intervals for these estimates were based on the binomial distribution.

## Results

**Figure 1:**
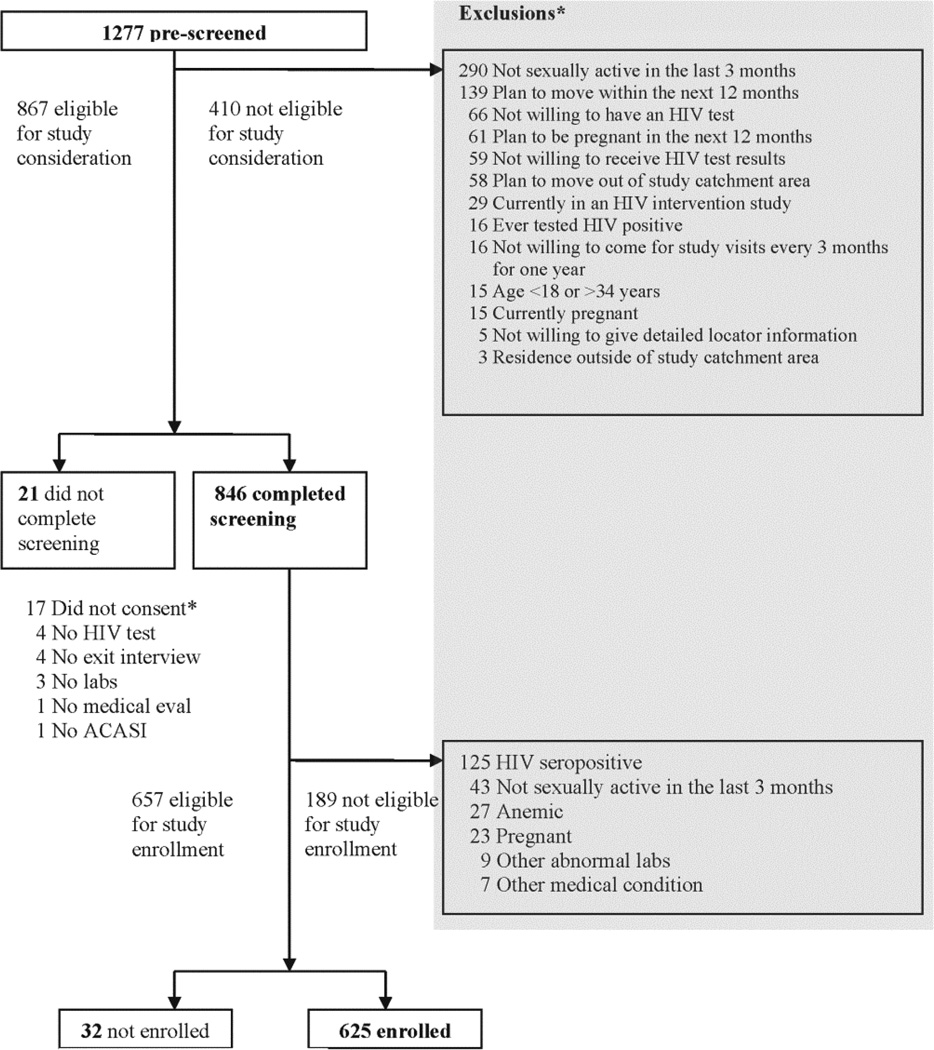
Kisumu Incidence Cohort Study profile at baseline screening and enrollment

Between March 2007 and March 2008, a total of 1,277 individuals were prescreened for basic study eligibility, of whom 867 (68%) met preliminary criteria (Figure 1). Among the 410 (32%) individuals initially excluded, reasons for the exclusions included sexual inactivity (n = 290, 70.7%), intention to move away from Kisumu (n = 139, 33.9%), intention to become pregnant (n = 61, 14.9%), and unwillingness to undergo HIV testing [n = 66 (38 male + 28 female; p = NS), 16.1%] or receive HIV test results [n = 59 (39 male + 20 females; p = 0.10), 14.4%]. A few were excluded for other reasons such as current enrollment in an HIV intervention study (n = 29, 7.1%), previously tested HIV positive (n = 16, 3.9%), unwillingness to come for study visits (n = 16, 3.9%) and current pregnancy (n = 15, 3.7%). Among the 846 who met the criteria and completed the screening, 625 (48.9% of those recruited) were enrolled; 189 were not eligible after completing the ACASI questionnaire and laboratory testing. See Figure 1 for additional details regarding baseline screening and enrollment.

### HIV prevalence among individuals presenting for enrollment

Among those who completed screening, overall HIV prevalence was 14.8% (125/846); 21.5% among females (91/424) and 8.1% among males (34/422). Prevalence was significantly higher among females than males overall and in all age groups with the exception of the 30 to 34 years age group (Table 2). In addition, there was a significant difference in overall HIV prevalence among the three age groups (χ^2^(2) = 29.2; p < 0.001). Pairwise comparisons showed that the 25 to 29 years (χ^2^(1) = 10.8; p < 0.001) and the 30 to 34 years age groups (χ^2^(1) =24.8; p <0.001) had higher prevalence than the 18 to 24 years age group.

### Enrolled compared to non-enrolled individuals: demographic characteristics

Differences were found in some demographic characteristics between enrolled and not enrolled persons, specifically in terms of age, gender, marital status, education, language preference, and migration history (Table 3). Compared to individuals not enrolled in the study, a greater proportion of individuals who were enrolled were younger (18 to 24 years) (p = 0.006), male (p < 0.001), single (p < 0.001), chose English as their ACASI language (p < 0.001), were a current student (p = 0.001), ever attended school (p = 0.005), completed a higher level of education (p < 0.001), and reported less than one week as their longest time out of town in the last three months (p = 0.002). In multivariate analysis, age, sex and language remained independently associated with enrollment. The odds of being enrolled were higher for persons 18 to 24 years compared to those 30 to 34 years of age [adjusted odds ratio (AOR) = 2.18, CI = 1.13, 4.21] and males compared to females [AOR = 2.07, CI = 1.43, 2.99]. The odds of being enrolled were lower for those who chose Dholuo for their ACASI language compared to English (AOR = 0.57, CI = 0.33, 0.99).

### Enrolled compared to non-enrolled individuals: motivation for participation, male circumcision, HIV and STIs

A lower proportion of persons enrolled versus not enrolled reported their primary motivation for participation was due to incentives provided by the study (p = 0.015), tested positive for HSV-2 (p < 0.001), gonorrhea (p = 0.014) and syphilis (p = 0.008). There was no significant difference in male circumcision or chlamydia (Table 3). All persons enrolled had to be HIV-1 negative.

### Enrolled males compared to enrolled females: sexual behaviors and other HIV risk behaviors

Overall, the most prevalent HIV risk behaviors were unprotected sex (49%), alcohol use (45%), and transactional sex (30%) in last the three months (Table 4). Compared to females, a greater proportion of males reported using any alcohol (p < 0.001) or any drug in the last three months (p < 0.001), a history of oral sex (p = 0.032), sex with partner other than a spouse or main partner in the last three months (p < 0.001), ever having a blood transfusion (p = 0.009), had an HIV test prior to screening (p < 0.042), and having knowledge of their last HIV test result (p = 0.016) (Table 4). Men also reported a younger age at sexual debut (p < 0.001), a greater number of lifetime partners (p < 0.001), a greater number of opposite sex partners in the last three months (p < 0.001), and also a greater number of times ever tested for an STI and number of lifetime HIV tests (p < 0.001).

Compared to males, a greater proportion of females reported being involved in a polygamous relationship (p = 0.026), a history of anal sex (p = 0.003), and a history of forced sex (p = 0.001).

## Discussion

Our analysis demonstrated the successful recruitment of young adults to assess HIV incidence and to prepare for future HIV prevention studies in this high HIV prevalence region of western Kenya. A total of 625 individuals were enrolled out of a total of 1,277 prescreened. Among the 846 individuals who completed screening, 14.8% HIV prevalence was found among persons of previously unknown status compared to 14.9% HIV prevalence (15 to 64 years) for this area of Kenya reported in 2007 [5]. Recent studies in nearby areas in western Kenya reported an HIV prevalence of 15.4% among individuals aged 13 to 34 years [11] and 14.3% among individuals 18 to 55 years [12]. In these studies, as in many previous studies [5,13], we found a higher HIV prevalence among females than males, and increasing prevalence with increasing age emphasizing the disproportionate burden of disease among girls and women in sub-Saharan Africa [14].

In contrast to other similar studies, our analysis assessed differences between individuals enrolled and those not enrolled. While there were many similarities between these two groups, there were a few differences. The odds of being enrolled were higher for younger (18 to 24 years) compared to older persons and for men compared to women, and lower for persons who chose Dholuo for their ACASI language. That the enrolled group was younger and predominantly male represents the epidemiology of the epidemic in this population of western Kenya, specifically in that older individuals and females are more likely to be infected [11,12,15]. Our ability to enroll younger persons prior to HIV infection, however, is desirable for implementation of future HIV prevention trials in this population.

The difference in language preference for ACASI may be attributed to the higher proportion of people speaking English in the urban area where the study was conducted as compared to Dholuo being preferred more in the rural areas. Two additional results are worth noting. First, among all men screened, 46% were circumcised as determined by clinical examination. This is more than the 27.5% reported almost a decade earlier [16] and is expected considering the results of a study conducted in Kisumu, showing the protective effects of male circumcision for HIV infection [7]. Second, among individuals presenting for enrollment in our study, about 30% were excluded because they did not want an HIV test or did not want to know their results, perhaps due to concerns about potential negative social repercussions of being HIV infected, which has been reported in other studies [11,17]. Hopefully these fears of stigmatization will decrease with greater access to HIV treatment and other services in recent years so that HIV will be considered more of a chronic disease.

We also confirmed gender differences in sexual behaviors and other HIV risk behaviors that have been reported previously [13,18]. In addition to reporting an earlier age at sexual debut and a greater number of partners than women, men more frequently reported such risk behaviors as using alcohol, using recreational drugs, having sex with a partner other than a spouse or main partner, and having ever been treated for an STI. Problem alcohol drinking has been reported to be higher among men than women [19]. In addition, having sex while intoxicated has been associated with high risk of sexual behaviors such as unprotected sex with casual partners and paying for sex [20]. The 2000–2001 UNAIDS prevention campaign, titled “Men Make a Difference”, attempted to address this issue and worked to motivate men and women to communicate about HIV risk behaviors such as alcohol and drug use and to encourage men to care for themselves and their partners and families [21].

The higher HIV prevalence among women not eligible for enrollment is likely due to multiple factors including HIV transmission being more efficient from infected men to uninfected women [22], young girls having sex with older men in exchange for gifts or favors [23], early marriage of adolescent girls which can lead to other health problems through early pregnancy [24], and the loss of educational opportunities [13,25,26]. Education is an effective means of HIV prevention in that it equips children to make informed decisions about their own health and at the same time contributes to the empowerment and economic independence of girls and women [27]. Other gender differences, such as unwanted or forced sex can be explained by the subordinate position of women in the society where many girls and women lack not only resources, but the ability to obtain resources, the ability to obtain leadership positions, and the power to make decisions [15,28]. It is noteworthy that there was no significant difference between men and women in the proportion reporting receiving food, shelter, money, gifts or other favors for sex in the last three months given that a key HIV risk factor in the literature is payment to girls and women for sex (*e.g*., Sugar Daddy) [29–31]. The literature is sparse regarding payment to boys/men for sex (“Sugar Mummies”), but gifts or favors from girls to boys has been reported as part of the dating ritual [30].

Our analysis findings should be interpreted in light of at least two potential limitations. First, our research participants were self-selected and may not be representative of the Kisumu population. Second, there may be response bias as with all surveys although we believe use of ACASI facilitated more honest responding [32]. The strength of our analysis, however, is that it provided information on both participants enrolled in the study and individuals who were not enrolled and the reasons that they were not enrolled.

In conclusion, in an area of Kenya with very high HIV prevalence, we successfully enrolled persons with HIV risk characteristics in an HIV incidence cohort. Overall, the enrolled and not enrolled groups were comparable with a few exceptions such as differences in age and gender. Gender-related differences in sexual behaviors and other HIV risk behaviors were similar to those previously reported in Kenya. Notably, females had higher HIV prevalence (21.5% versus 8.1%). This finding underscores the importance of focusing on adolescent girls and women to combat the HIV epidemic [33,34] via biomedical interventions such as microbicides [35] and/or structural/policy interventions such as education [36] which would provide girls with increased access to HIV knowledge, economic resources, and decision-making power [37].

## Figures and Tables

**Table 1:** Inclusion and exclusion criteria for the Kisumu Incidence Cohort Study, Kisumu, Kenya, 2007–2008

**Inclusion criteria**
Male and female residents of the Kisumu catchment area
Age 18 to 34 years
Engagement in sexual activity ≥ one time within the past 3 months
HIV seronegative by licensed rapid HIV testing in parallel
Available for 12 months of participation in the study
Able and willing to provide informed consent
Able to provide detailed locator information to ensure adequate, timely follow-up
Meet the following laboratory criteria: serum creatinine < 1.5mg/dl, haemoglobin ≥ 9.0g/dl, platelets > 50,000/ml, ALT < 2.5 times the upper limit of normal
Willing to comply with study procedures and requirements if meet criteria for study eligibility
**Exclusion criteria**
HIV seropositive
Pregnant or plan to become pregnant in next 12 months if female
Plan to reside outside the Kisumu catchment area for >3 months
Evidence of clinically significant cardiac, respiratory, hepatic, gastrointestinal, endocrine, hematologic, psychiatric, neurologic, or allergic disease that would compromise the ability of the participant to provide competent informed consent, or to complete study procedures or study requirements as determined by the principal investigator or designated associate.
The clinical significance of any abnormality is to be evaluated in the context of the safety of the patient volunteer and the objectives of this study.
Active participation in HIV intervention studies that might influence HIV incidence or risk behaviour

**Table 2:** Baseline prevalence of HIV from the Kisumu Incidence Cohort Study, Kisumu, Kenya, 2007–2008, by gender and age group

	Males (N=422)		Females (N=424)			Overall	
Age (years)	Prevalence % (n)	95% CI	Prevalence % (n)	95% CI	p-value	Prevalence % (n)	95% CI
18–24	5.1 (16)	(3.0, 8.1)	17.4 (56)	(13.4, 22.0)	<0.001	11.3 (72)	(9.0, 14.1)
25–29	13.2 (10)	(6.5, 22.9)	30.1 (22)	(19.9, 42.0)	0.016	21.5 (32)	(15.2, 28.9)
30–34	24.2 (8)	(11.1, 42.3)	44.8 (13)	(26.5, 64.3)	0.111	33.9 (21)	(22.3, 47.0)
Overall	8.1 (34)	(5.6, 11.1)	21.5 (91)	(17.7, 25.7)	<0.001	14.8 (125)	(12.5, 17.4)

**Table 3:** Baseline characteristics of persons screened for the Kisumu Incidence Cohort Study, Kisumu, Kenya, 2007–2008, by enrollment status (N = 846)

Characteristic	Screened and enrolled (N=625)^a^ n (%)	Screened and not enrolled (N=221)^a^ n (%)	p-value	Total screened (N=846)^a^ n(%)
**Demographic characteristics**				
Age range (years)†			0.006	
18–24	485 (78)	150 (68)		635 (75)
25–29	103 (16)	46 (21)		149 (17)
30–34	37 (6)	25 (11)		62 (7)
Gender†			<0.001	
Female	278 (44)	146 (66)		424 (50)
Male	347 (56)	75 (34)		422 (50)
Marital status			<0.001	
Single/Never married	407 (65)	108 (50)		515 (61)
Not married, but living as married	55 (9)	17 (8)		72 (9)
Married	144 (23)	70 (32)		214 (25)
Separated/Divorced	14 (2)	14 (6)		28 (3)
Widowed	4 (1)	9 (4)		13 (2)
District of residence			0.831	
Kisumu	611 (98)	218 (99)		829 (98)
Vihiga	6 (1)	1(0)		7 (1)
Nyando	8 (1)	2 (1)		10 (1)
Ethnic group or tribe			0.336	
Luo	527 (84)	182 (83)		709 (84)
Luhya	55 (9)	25 (11)		80 (9)
Kisii	28 (4)	5 (2)		33 (4)
Kikuyu	5 (1)	4 (2)		9 (1)
Maasai	1 (0)	0 (0)		1 (0)
Other	9 (1)	4 (2)		13 (2)
Language chosen for ACASI interview†			<.001	
English	352 (56)	76 (34)		428 (51)
Kiswahili	82 (13)	41 (19)		123 (15)
Dholuo	191 (31)	104 (47)		295 (35)
Religion			0.153	
Roman Catholic	226 (36)	92 (42)		318 (38)
Protestant/other Christian	290 (46)	80 (36)		370 (44)
Muslim	21 (3)	6 (3)		27 (3)
Nomiya^b^	31 (5)	14 (6)		45 (5)
No religion	17 (3)	8 (4)		25 (3)
Other	40 (6)	20 (9)		60 (7)
Education				
Currently a student- n (%) yes	187 (30)	41 (19)	0.001	228 (27)
Ever attended school- n (%) yes	601 (96)	202 (92)	0.005	803 (95)
Highest level of schooling completed			<0.001	
No school	22 (4)	18 (8)		40 (5)
Primary	136 (22)	78 (35)		214 (25)
Secondary	240 (39)	71 (32)		311 (37)
Technical training	51 (8)	12 (5)		63 (7)
College	160 (26)	40 (18)		200 (24)
University	13 (2)	1 (0)		14 (2)
Employment				
Currently working	296 (47)	108 (49)	0.673	404 (48)
Main type of work			0.823	
No work	328 (53)	112 (51)		440 (52)
Farmer	22 (4)	6 (3)		28 (3)
Salaried	17 (3)	5 (2)		22 (3)
Casual worker	87 (14)	30 (14)		117 (14)
Self-employed	101 (16)	45 (21)		146 (17)
Homemaker	46 (7)	16 (7)		62 (7)
Other	21 (3)	5 (2)		26 (3)
Earned < minimum wage in the last 30 Days^c^	568 (94)	194 (94)	0.944	762 (94)
**Migration history**				
Moved for ≥ 3 months within past 2 years- n (%) yes	194 (31)	68 (31)	0.940	262 (31)
Moved within the last 3 months- n (%) yes	72 (12)	30 (14)	0.420	102 (12)
Longest time out of town in the last 3 months			0.002	
None	189 (31)	93 (44)		282 (34)
<1 week	236 (39)	56 (27)		292 (36)
Between 1 week and 1 month	89 (15)	30 (14)		119 (14)
>1 month	96 (16)	32 (15)		128 (16)
**Motivation for participation**				
Free medical care for sexually transmitted infections and common illness- n (%) yes	490 (79)	178 (81)	0.431	668 (79)
Incentives- n (%) yes	228 (37)	101 (46)	0.015	329 (39)
**Circumcision, HIV and Sexually Transmitted** **Infections**				
Circumcised (males only) - n (%) yes	161 (47)	28 (38)	0.142	189 (46)
HIV-1 - n (%) positive HSV-2 – n (%) positive	0 (0) 141 (24)	125 (57) 105 (53)	N/A <0.001	125 (15) 246 (32)
Chlamydia – n (%) positive	19 (3)	5 (2)	0.550	24 (3)
Gonorrhea- n(%) positive Syphilis – n (%) positive	10 (2) 6 (1)	10 (5) 8 (4)	0.014 0.008	20 (2) 14 (2)

a N for certain individual questions and laboratory results do not add up to total as some participants had missing data

b Religion of the predominant Luo ethnic group in the area (38)

c Less than Kenya Shillings 6130 earned in the last 30 days

† Independently associated with enrollment in multivariable analysis.

**Table 4:** Baseline characteristics of persons enrolled into the Kisumu Incidence Cohort Study, Kisumu, Kenya, 2007–2008, by gender (N = 625)

Characteristic	Males enrolled (N=347)	Females enrolled (N=278)	p-value	Overall enrolled (N=625)
**Family characteristics**				
Head of household - n (%) yes	125 (36)	73 (26)	<0.001	198 (32)
Parent - n (%) yes	103 (31)	148 (55)	<0.001	251(42)
**Partnership characteristics**				
>1 wife or co-wife at present - n (%) yes	2 (1)	8 (3)	0.026	10 (2)
History of ever inheriting a wife/being inherited† - n (%) yes	13 (4)	14 (5)	0.444	27 (4)
**Alcohol and drug use in the last 3 months**				
Any alcohol use - n (%) yes	205 (59)	76 (27)	<0.001	281 (45)
Any recreational drug use - n (%) yes	98 (28)	11 (4)	<0.001	109 (18)
Any injecting drug use - n (%) yes	5 (1)	2 (1)	0.469	7 (1)
**Sexual history**				
- Age at sexual debut – median (IQR)	15 (13–18)	17 (16–19)	<0.001	16 (15–18)
-Number of lifetime sex partners- median (IQR)	6 (3–10)	3 (2–4)	<0.001	4 (2–8)
History of anal sex - n (%) yes	54 (16)	69 (25)	0.003	123 (20)
History of heterosexual oral sex - n (%) yes	99 (29)	58 (21)	0.032	157 (25)
History of forced sex - n (%) yes	45 (13)	65 (23)	<0.001	110 (18)
**Sexual risk behavior in the last 3 months**				
- Number of opposite sex sexual partners- median (IQR)	2 (1–3)	1 (1–2)	<0.001	1 (1–2)
-Had sex partner other than spouse or main partner - n (%) yes	181 (53)	60 (22)	<0.001	241 (39)
-Condom use last time had sex with spouse or main partner n(%) no	158 (46)	147 (53)	0.156	305 (49)
-Thinks spouse or main partner had sex with both men and women - n (%) yes	14 (4)	13 (5)	0.697	27 (4)
Forced sex - n (%) yes	29 (8)	37 (13)	0.046	66 (11)
Exchanged sex┼ (n (%) yes	114 (33)	73 (26)	0.074	187 (30)
**Non-sexual risk characteristics**^				
Ever had a blood transfusion- n (%) yes	65 (19)	31 (11)	0.009	96 (15)
Ever had scarification - n (%) yes	46 (13)	45 (16)	0.300	91 (15)
Ever been treated for a sexually transmitted infection - n (%) yes	65 (19)	23 (8)	<0.001	88 (14)
Treated for an STI in the last 3 months - n (%) yes	16 (5)	5 (2)	0.053	21 (3)
**HIV testing history and attitudes**				
Ever had an HIV test prior to screening - n (%) yes	250 (72)	179 (65)	0.042	429 (69)
- Lifetime number of times ever tested for HIV- median (IQR)	2 (0–3)	1 (0–3)	<0.001	1 (0–3)
Knows result of last HIV test – n (%) yes	221 (64)	151 (55)	0.016	372(60)

† “Inherited” refers to the cultural practice of the Luo, the predominant ethnic group in the area, where a widow is inherited by the male next of kin of the deceased husband (39).

┼ includes exchanging sex for shelter, food, money, and/or gifts/other favors.

^ Circumcisions status was not compared because it was clinically determined for males and self-reported for females.
